# Rice body synovitis of the wrist mimicking tuberculosis in an HIV-positive patient: A case report

**DOI:** 10.1097/MD.0000000000048139

**Published:** 2026-03-27

**Authors:** Bo Zhou, Shanpeng Luo, Xin Yan, Chunyuan Liao, Jiayi Feng, Yangyang Chen

**Affiliations:** a Third Department of Surgery (Traumatic Orthopedics), Guiyang Public Health Clinical Center, Guiyang, China; b Anesthesia Operating Room, Guiyang Public Health Clinical Center, Guiyang, China; c Department of Pathology, Guiyang Public Health Clinical Center, Guiyang, China.

**Keywords:** chronic synovitis, histopathology, immunocompromised host, rice body synovitis, tuberculosis, wrist

## Abstract

**Rationale:**

Rice body synovitis is a rare benign synovial disorder with an insidious onset and nonspecific clinical features that often lead to diagnostic uncertainty or delay.

**Patient concerns:**

A 68-year-old woman with human immunodeficiency virus infection and a history of cured pulmonary tuberculosis presented with right wrist pain and swelling for 3 years, which had worsened over the preceding 2 months. She also reported limited motion and a palpable mass.

**Diagnoses:**

Preoperative imaging showed moth-eaten bone destruction, peri-articular abscesses, joint space narrowing, and intra-articular rice bodies, raising a strong suspicion of tuberculous arthritis. However, intraoperative assessment and histopathological analysis confirmed rice body synovitis and found no evidence of *Mycobacterium tuberculosis* infection or granulomatous inflammation.

**Interventions:**

Surgical exploration and synovectomy were performed, various intra-articular bodies were removed, and the affected tissues were debrided.

**Outcomes:**

The patient recovered well after surgery, with significant symptom relief and improved joint function. No recurrence was observed during 8 months of follow-up.

**Lessons:**

In regions where tuberculosis is prevalent, clinicians should avoid diagnosing tuberculous synovitis solely based on intra-articular bodies or radiographic abnormalities, even in high-risk patients. Histopathological evaluation is essential for definitive diagnosis and appropriate management.

## 1. Introduction

Rice body synovitis (RBS) is a chronic proliferative synovial disorder characterized by the formation of numerous small, rice-like loose bodies within joint cavities, bursae, or tendon sheaths. Its precise pathogenesis remains unclear, but chronic inflammation is believed to play a central role. Patients usually present with gradually progressive joint swelling, palpable masses, and restricted function. The condition most commonly affects large joints such as the shoulder and knee,^[1-4]^ whereas involvement of the wrist is uncommon.^[5]^

We report the case of a 68-year-old woman with human immunodeficiency virus (HIV) infection and a history of treated pulmonary tuberculosis who developed chronic swelling, pain, and limited motion of the right wrist. Preoperative imaging demonstrated moth-eaten bone destruction, surrounding abscess formation, joint space narrowing, and intra-articular rice bodies, strongly suggesting tuberculous arthritis. However, postoperative histopathology confirmed RBS and showed no evidence of *Mycobacterium tuberculosis* infection or granulomatous inflammation. This case is important for its rare wrist involvement and for occurring in a patient with both HIV and previous tuberculosis. The radiologic findings closely resembled those of tuberculous arthritis and posed a substantial diagnostic challenge. By presenting this case alongside a review of relevant literature, we aim to improve clinical, radiologic, and pathologic recognition of RBS, enhance diagnostic accuracy, and help clinicians avoid unnecessary antitubercular therapy or excessively aggressive surgical management in similarly complex presentations.

## 2. Case presentation

### 2.1. Clinical presentation

A 68-year-old woman was admitted on February 26, 2024, with a 3-year history of right wrist pain that had worsened during the preceding 2 months and was associated with a palpable mass. The symptoms began insidiously and progressed despite conservative treatment, including topical herbal remedies and acupuncture. During the 2 months before admission, the pain became more severe, and wrist mobility declined significantly. A prior computed tomography (CT) scan performed at another institution showed multiple osteolytic lesions and surrounding soft tissue swelling in the right wrist, raising suspicion for tuberculosis or gout.

Her medical history included HIV infection diagnosed 5 years earlier and controlled with regular antiretroviral therapy, pulmonary tuberculosis successfully treated 3 years earlier, and hypertension that had remained well controlled for 1 year. She reported no history of rheumatoid arthritis or gout.

Physical examination demonstrated diffuse swelling of the right wrist without erythema or increased local temperature. We palpated a soft, poorly defined, fluctuant mass measuring approximately 3 cm in diameter. It was tender on pressure and associated with significant limitation of wrist flexion and extension. Distal perfusion and sensation of the hand remained intact.

Laboratory testing showed T-lymphocyte subset counts as follows: cluster of differentiation 3-positive T cells 794 cells/μL, cluster of differentiation 3-positive/cluster of differentiation 4-positive T cells 212 cells/μL, and cluster of differentiation 3-positive/cluster of differentiation 8-positive T cells 580 cells/μL, yielding a cluster of differentiation 4/cluster of differentiation 8 ratio of 0.36. The erythrocyte sedimentation rate was 19 mm/h, while the white blood cell count and C-reactive protein level were within normal ranges. Rheumatologic screening results were unremarkable. The T-spot test for tuberculosis assay and smear, culture, and nucleic acid amplification testing of wrist joint aspirates were all negative for *M tuberculosis*.

### 2.2. Imaging findings

**Figure 1.**
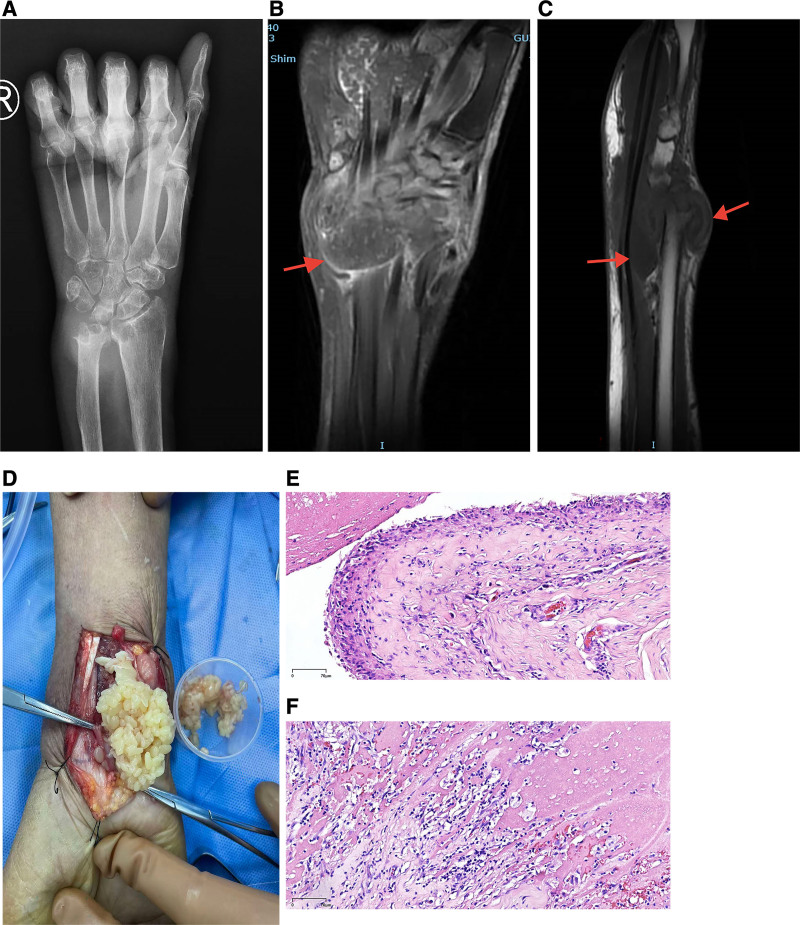
(A) Plain radiograph of the right wrist demonstrating indistinct joint spaces and moth-eaten bone destruction of the distal radius and ulna. (B) Coronal proton density–weighted MRI with fat suppression showing multiple granular signal abnormalities (red arrows) within the flexor tendon sheath. (C) T1-weighted fast spin-echo image revealing well-circumscribed hypointense masses surrounding the ulnar styloid and within the distal radioulnar joint (red arrows). (D) Intraoperative photograph demonstrating numerous rice bodies within the lesion. (E, F) Histopathology: microscopy shows well-defined rice bodies and hyperplastic synovium with stromal lymphocytic infiltration and focal vascular proliferation (Hematoxylin and Eosin × 100). The bodies consist of acellular, eosinophilic, hyalinized collagen lacking cartilaginous elements and occasionally containing scattered lymphocytes (Hematoxylin and Eosin × 100). MRI = magnetic resonance imaging.

Chest CT revealed fibrotic scarring and nodular opacities consistent with healed pulmonary tuberculosis. Plain wrist radiographs showed joint space obscuration and destructive changes in the distal radius and ulna, accompanied by adjacent soft tissue swelling (Fig. 1A). Magnetic resonance imaging (MRI) demonstrated poorly defined, “moth-eaten” bone destruction involving the carpal bones, distal radius and ulna, and the proximal second and third metacarpals, together with extensive soft tissue edema (Fig. 1B, C).

Given the patient’s HIV status, previous tuberculosis, and imaging findings suggestive of tuberculous arthritis, surgical exploration was carried out for both diagnostic and therapeutic purposes. This approach allowed definitive histopathologic evaluation while simultaneously addressing progressive structural damage and severe symptoms.

### 2.3. Surgical procedure

Pronounced swelling was present in the right wrist and distal forearm, with prominence of the distal ulna. We made an approximately 8-cm S-shaped incision extending from the ulnar aspect of the distal forearm to the volar wrist. After opening the tendon sheath, numerous yellowish, rice-shaped loose bodies were released (Fig. 1D). No caseous necrosis was identified.

The superficial flexor tendons were partially avulsed and adherent, so the affected portions were carefully separated and excised while preserving the median nerve. The pronator quadratus muscle and joint capsule appeared dark gray and were partially torn. The ulnar collateral and radioulnar ligaments were lax and ruptured, resulting in dislocation of the distal radioulnar joint. We observed destructive changes in the carpal bones and distal ulna, and the cartilage surface detached easily from the underlying bone. All necrotic tissue, synovium, and debris were then thoroughly debrided, the remaining capsule and ligaments were repaired, the joint was reduced, and it was immobilized with a plaster cast.

### 2.4. Pathological examination

The excised loose bodies and adjacent synovial tissue were submitted for histopathological evaluation. Microscopic examination showed classic features of RBS, including well-circumscribed structures composed of acellular, eosinophilic, hyalinized collagen without cartilaginous elements and with occasional scattered lymphocytes. The surrounding synovium showed hyperplasia, stromal lymphocytic infiltration, and focal vascular proliferation. No granulomatous inflammation was observed. These findings supported a diagnosis of RBS (Fig. 1E, F).

### 2.5. Postoperative follow-up

The patient’s pain and swelling improved significantly after surgery. We removed the sutures 2 weeks postoperatively and began supervised rehabilitation exercises. At the 6-week follow-up, wrist flexion and extension had improved substantially, and there was no recurrent swelling or tenderness. Radiographs obtained 8 months after surgery showed no evidence of lesion recurrence. The chronological order of key clinical events is summarized in Table S1, Supplemental Digital Content, https://links.lww.com/MD/R583. A timeline of the key clinical events is also provided in Table 1.

## 3. Discussion

### 3.1. Pathogenesis

Rice bodies were first documented by Riese in 1895 in a patient with tuberculous arthritis of the knee and were later described in the English-language literature by Rogers in 1927.^[6]^ Since that time, multiple hypotheses have been proposed to explain their pathogenesis. Suggested mechanisms include chronic intra-articular synovitis leading to fibrin aggregation and synovial tissue necrosis,^[7]^ microinfarction caused by localized ischemia,^[8]^ and alterations in synovial fluid viscosity or composition that favor fibrin encapsulation.^[9]^ Although early reports suggested that rice bodies formed exclusively within synovial tissue, subsequent observations of extra-articular occurrences indicate that they may also develop independently of the synovium.^[10]^

In this case, RBS affected the wrist. During surgery, numerous rice bodies adherent to both the joint capsule and the synovial membrane were observed, supporting a synovial origin, consistent with previous reports.^[11]^ RBS is an uncommon condition with heterogeneous causes. Current evidence indicates that it may arise secondary to chronic inflammatory disorders, particularly rheumatoid arthritis and tuberculous infection.^[12-14]^

### 3.2. Clinical presentation

RBS commonly presents with joint swelling, pain, and stiffness and may cause recurrent effusions or mechanical symptoms, such as locking.^[6]^ When the wrist is involved, the lesion can exert mass effect on adjacent structures and, in some cases, compress the median nerve, leading to carpal tunnel syndrome.^[15]^ In comparison, tuberculous arthritis of the wrist typically occurs in younger or middle-aged adults and progresses slowly with insidious onset of swelling, pain, and restricted motion. Because these symptoms lack specificity, diagnosis is often delayed. In advanced disease, extensive bone destruction and joint deformity may result in functional limitation or ankylosis.^[16]^ The histopathological hallmark of articular tuberculosis is granulomatous inflammation, frequently accompanied by intra-articular rice bodies.^[17]^ In this case, the patient had an HIV infection and a previous history of pulmonary tuberculosis. Individuals with HIV-related immunodeficiency are particularly susceptible to opportunistic infections, among which tuberculosis is common.^[18]^ HIV-associated immune dysfunction facilitates *M tuberculosis* infection, disease progression, and transmission.^[19]^ Given the established association between tuberculosis and intra-articular rice body formation, tuberculous arthritis was strongly suspected before surgery.

### 3.3. Differential diagnosis

Accurate pretreatment differentiation of RBS from other proliferative synovial disorders is essential, particularly from synovial chondromatosis and pigmented villonodular synovitis.^[20-22]^ Imaging plays a central role in this process. Because rice bodies consist mainly of fibrin, they are usually radiolucent and not visible on plain radiographs. On MRI, they typically appear as iso- to hypointense foci on both T1- and T2-weighted sequences,^[7,23]^ scattered within hyperintense synovial fluid and producing the characteristic “sesame seed” or “snowstorm” pattern.^[16]^ The imaging findings in this patient matched these classic features.

In synovial chondromatosis, loose bodies contain cartilaginous components that may calcify or ossify, allowing detection on radiographs or CT as multiple nodular high-density lesions of varying size. Advanced disease may show joint space narrowing and cortical erosion due to pressure effects. MRI appearance varies with lesion maturation: cartilaginous nodules display typical cartilage signal intensity, whereas ossified nodules show a central short-T1, long-T2 signal surrounded by peripheral calcification with long-T1, short-T2 characteristics.^[24-26]^. Pigmented villonodular synovitis can be distinguished from RBS by hypointense MRI regions produced by hemosiderin deposition and susceptibility artifacts on gradient-echo sequences, findings absent in RBS.^[27]^ Currently, MRI is the preferred modality for detecting and characterizing rice body formation.^[28]^

In this case, coronal proton density–weighted MRI demonstrated clustered granular signals of low to intermediate intensity consistent with aggregates of rice bodies. These lesions appeared faint or barely visible on T1-weighted images, reflecting the typical MRI profile of fibrin-rich tissue with relatively low water content. These features enabled clear differentiation from synovial chondromatosis and pigmented villonodular synovitis.^[22]^ Other causes were considered but found to be less likely. Rheumatoid arthritis-related synovitis was unsupported by negative serologic tests and the absence of systemic inflammatory manifestations. Gout was unlikely because of the patient’s clinical history and normal uric acid levels. In addition to typical intra-articular bodies, imaging revealed moth-eaten bone destruction with surrounding abscess formation and joint space narrowing. Based on these findings, tuberculous arthritis was prioritized as the leading preoperative diagnosis. When clinical and radiologic features overlap, however, histopathological evaluation remains the most reliable method for definitive distinction.

### 3.4. Management

At present, no universally accepted treatment guideline exists for RBS. Although its exact pathogenesis remains uncertain, intra-articular rice bodies can perpetuate inflammation by acting as persistent mechanical and inflammatory stimuli within the joint. Multiple studies have shown that surgical removal of these bodies, combined with synovectomy, effectively relieves symptoms and reduces local inflammatory activity.^[29-31]^ Surgeons may use either an open or an arthroscopic approach for excision. In this case, the patient showed significant postoperative improvement and no recurrence during follow-up.

A key therapeutic challenge in wrist RBS is identifying the underlying cause, which may include tuberculous infection, rheumatoid arthritis, or idiopathic tenosynovitis. Therefore, optimal management requires not only surgical treatment but also targeted therapy for the primary condition and careful control of systemic inflammation to minimize the risk of recurrence.

### 3.5. Limitations

This case report has several limitations. First, it describes a single patient, which restricts the generalizability of the observations. Larger case series or controlled studies are required to confirm the diagnostic challenges and management considerations outlined here. Second, although the 8-month follow-up demonstrated excellent short-term outcomes, it is too brief to evaluate long-term recurrence or delayed complications. Finally, the diagnosis was based on routine histopathology; molecular or immunohistochemical analyses of the excised bodies were not performed and might have provided further insight into their composition and pathogenesis.

### 3.6. Clinical implications

This report describes a rare case of RBS affecting the wrist. The presentation is noteworthy because the patient was an older adult with HIV infection and a previous history of successfully treated pulmonary tuberculosis, both factors that increased her risk for tuberculous arthritis. Preoperative clinical evaluation and imaging strongly supported this diagnosis, with evidence of bone destruction, joint space narrowing, and intra-articular bodies. However, postoperative histopathological analysis demonstrated findings consistent with RBS and no granulomatous inflammation, excluding tuberculosis.

This discrepancy highlights an important diagnostic principle: the presence of rice bodies alone is not pathognomonic for tuberculous synovitis. Although the mechanism underlying chronic inflammation and body formation in this patient remains unclear, the diagnostic course offers a valuable clinical lesson. In complex cases, especially in immunocompromised individuals with previous tuberculosis living in endemic regions, clinicians should avoid equating rice body formation automatically with tuberculous synovitis. Careful differential diagnosis and histopathological confirmation are essential. Adhering to this principle can reduce misdiagnosis and ensure appropriate management in similarly challenging situations. Further investigation into the etiology of RBS is warranted to clarify its pathogenesis and support more targeted therapeutic strategies.

**Table 1:** Timeline of key events.

Time point	Event	Key findings/procedures
Approximately 2021	Symptom onset	Insidious onset of pain and swelling in the right wrist
Before February 2024	External imaging	External computed tomography scan revealed multiple osteolytic lesions in the right wrist, raising suspicion of tuberculosis or gout
February 26, 2024	Hospital admission	Admitted due to worsening symptoms (pain, palpable mass, restricted motion)
Post-admission	Preoperative evaluation	Laboratory tests: T-spot test for tuberculosis and other tuberculosis-related assays were negative. Imaging: wrist X-ray and magnetic resonance imaging showed moth-eaten bone destruction, joint space narrowing, and rice bodies; chest computed tomography indicated old pulmonary tuberculosis sequelae
	Surgical intervention	Surgical exploration and synovectomy were performed, with removal of extensive rice bodies and debridement of affected tissues
2 wk post-op	Routine post-discharge follow-up	Suture removal and initiation of functional exercise
6 wk post-op	Short-term follow-up	Marked improvement in wrist flexion and extension, with no swelling or tenderness
8 mo post-op	Mid-term follow-up	Imaging studies showed no evidence of recurrence

## Author contributions

**Data curation:** Bo Zhou.

**Formal analysis:** Xin Yan.

**Investigation:** Jiayi Feng, Yangyang Chen.

**Methodology:** Chunyuan Liao, Yangyang Chen.

**Resources:** Jiayi Feng.

**Software:** Shanpeng Luo.

**Supervision:** Yangyang Chen.

**Validation:** Chunyuan Liao.

**Visualization:** Yangyang Chen.

**Writing – original draft:** Bo Zhou, Shanpeng Luo.

**Writing – review & editing:** Bo Zhou, Shanpeng Luo, Xin Yan, Chunyuan Liao.

## Supplementary Material


