# Supplementary material for: Rice body synovitis of the wrist mimicking tuberculosis in an HIV-positive patient: A case report
Source: Medicine (Baltimore). 2026 Mar 27;105(13):e48139. doi: 10.1097/MD.0000000000048139 (PMC13034949; doi:10.1097/MD.0000000000048139)
Supplement: Supplementary file 1 [file medi-105-e48139-s001.docx]

**Table 1: Timeline of Key Events**

| Time Point | Event | Key Findings/Procedures |
| --- | --- | --- |
| Approximately 2021 | Symptom Onset | Insidious onset of pain and swelling in the right wrist. |
| Before February 2024 | External Imaging | External computed tomography scan revealed multiple osteolytic lesions in the right wrist, raising suspicion of tuberculosis or gout. |
| February 26, 2024 | Hospital Admission | Admitted due to worsening symptoms (pain, palpable mass, restricted motion). |
| Post-admission | Preoperative Evaluation | Laboratory tests: T-Spot Test for tuberculosis and other tuberculosis-related assays were negative. Imaging: wrist X-ray and magnetic resonance imaging showed moth-eaten bone destruction, joint space narrowing, and rice bodies; chest computed tomography indicated old pulmonary tuberculosis sequelae. |
|  | Surgical Intervention | Surgical exploration and synovectomy were performed, with removal of extensive rice bodies and debridement of affected tissues. |
| 2 weeks post-op | Routine post-discharge follow-up | Suture removal and initiation of functional exercise. |
| 6 weeks post-op | Short-term Follow-up | Marked improvement in wrist flexion and extension, with no swelling or tenderness. |
| 8 months post-op | Mid-term Follow-up | Imaging studies showed no evidence of recurrence. |
